# Matricide in psychotic patients: About 3 case reports

**DOI:** 10.1192/j.eurpsy.2021.1435

**Published:** 2021-08-13

**Authors:** S. Kolsi, S. Hentati, I. Baati

**Affiliations:** Psychiatry A, CHU Hédi Chaker Sfax, Sfax, Tunisia

## Abstract

**Introduction:**

Matricide is the murder of the mother and it is one the rarest reported homicides with rates varying between 1% and 4% of all murders. In our country, few studies have focused on this issue.

**Objectives:**

To precise circumstances of matricide in psychotic patients and the offender and victim’s profiles.

**Methods:**

We report three clinical cases who were hospitalized in the department of Psychiatry “A” at the Hedi Chaker university hospital in Sfax, Tunisia, because of non-judicial proceedings for dementia during the year of 2019.

**Results:**

Among 3 cases, two patients were male and they were aged respectively 26 and 48 years old. The third was female. All of them had very low educational and income levels and they lived with the victim. In 2 cases, the victim-offender relationship was conflictive and there was already exhibited violent behavior towards victim.Two patients had a psychiatric follow-up and many hospitalizations. The diagnoses were schizophrenia and schizoaffective disorder. However, it was a poor compliance and an interruption of treatment. In the third case, the medicolegal procedure was inaugural. His diagnosis was schizophrenia.All the victims were illiterate and unemployed. Their middle age was 64 years.In all cases, the crime was not premeditated, motivated by persecution delusions including the mother. It was committed in the family house while using a blunt object. The post homicide reaction was marked by coldness.

**Conclusions:**

Matricide has always been considered one of the most abhorrent crimes. Regular evaluation of psychotic patients’ dangerousness signals is needed to reduce the acting out.

**Conflict of interest:**

No significant relationships.

